# Should Artificial Intelligence-Based Patient Preference Predictors Be Used for Incapacitated Patients? A Scoping Review of Reasons to Facilitate Medico-Legal Considerations

**DOI:** 10.3390/healthcare13060590

**Published:** 2025-03-08

**Authors:** Pietro Refolo, Dario Sacchini, Costanza Raimondi, Simone S. Masilla, Barbara Corsano, Giulia Mercuri, Antonio Oliva, Antonio G. Spagnolo

**Affiliations:** 1 Research Centre for Clinical Bioethics & Medical Humanities, Università Cattolica del Sacro Cuore, Largo F. Vito 1, 00168 Rome, Italy; costanza.raimondi1@unicatt.it (C.R.); salvatoresimone.masilla@unicatt.it (S.S.M.); antoniogioacchino.spagnolo@unicatt.it (A.G.S.); 2 Department of Health Care Surveillance and Bioethics, Section of Bioethics and Medical Humanities, Università Cattolica del Sacro Cuore, Largo F. Vito 1, 00168 Rome, Italy; 3 Fondazione Policlinico Universitario A. Gemelli IRCCS, Largo F. Vito 1, 00168 Rome, Italy; barbara.corsano@unicatt.it (B.C.); antonio.oliva@unicatt.it (A.O.); 4 Department of Health Care Surveillance and Bioethics, Section of Legal Medicine, Università Cattolica del Sacro Cuore, Largo F. Vito 1, 00168 Rome, Italy; giulia.mercuri01@icatt.it

**Keywords:** artificial intelligence (AI), patient preference predictors (PPPs), surrogate decision-making, incapacitated patients

## Abstract

Background: Research indicates that surrogate decision-makers often struggle to accurately interpret and reflect the preferences of incapacitated patients they represent. This discrepancy raises important concerns about the reliability of such practice. Artificial intelligence (AI)-based Patient Preference Predictors (PPPs) are emerging tools proposed to guide healthcare decisions for patients who lack decision-making capacity. Objectives: This scoping review aims to provide a thorough analysis of the arguments, both for and against their use, presented in the academic literature. Methods: A search was conducted in PubMed, Web of Science, and Scopus to identify relevant publications. After screening titles and abstracts based on predefined inclusion and exclusion criteria, 16 publications were selected for full-text analysis. Results: The arguments in favor are fewer in number compared to those against. Proponents of AI-PPPs highlight their potential to improve the accuracy of predictions regarding patients’ preferences, reduce the emotional burden on surrogates and family members, and optimize healthcare resource allocation. Conversely, critics point to risks including reinforcing existing biases in medical data, undermining patient autonomy, raising critical concerns about privacy, data security, and explainability, and contributing to the depersonalization of decision-making processes. Conclusions: Further empirical studies are needed to assess the acceptability and feasibility of these tools among key stakeholders, such as patients, surrogates, and clinicians. Moreover, robust interdisciplinary research is needed to explore the legal and medico-legal implications associated with their implementation, ensuring that these tools align with ethical principles and support patient-centered and equitable healthcare practices.

## 1. Introduction

When a person who was once capable of making autonomous medical decisions loses that ability, the responsibility for making such choices typically shifts to others, often close family members or trusted associates. Ideally, the now-incapacitated individual (henceforth referred to as “the patient”) would have previously outlined their treatment preferences in an advance directive or a similar advance-care plan.

However, the reality is that most people do not make use of this option [1]. Moreover, even when an advance directive is available, it often fails to address the specific circumstances or complex medical dilemmas the patient may face. This places surrogate decision-makers in a challenging position. Moreover, such decisions are frequently made under significant emotional strain and in high-pressure, time-sensitive situations.

When in these difficult scenarios, the principles of “substituted judgment” and “best interests” are widely recognized as essential frameworks for surrogate decision-making [2]. Substituted judgment prioritizes making decisions based on what the patient would have chosen if they were able to decide. Conversely, the best interest approach focuses on selecting the option that best promotes the patient’s overall well-being.

Central to both frameworks is the importance of the patient’s preferences. The substituted judgment standard serves as a guide to identify what the patient would likely prefer in order to choose accordingly. Similarly, under the best interest principle, respecting an individual’s preferences is often considered strong evidence of—or even fundamental to—their well-being. Consequently, whether surrogate decisions are guided by substituted judgment or a best interest framework, decision-makers have ethical grounds to give considerable weight to the patient’s preferences in their deliberations.

Nonetheless, research [3] indicates that surrogate decision-makers often struggle to accurately interpret and reflect the preferences of the patients they represent. This discrepancy raises important concerns about the reliability of current surrogate decision-making practices.

To tackle these challenges, Wendler et al. [4] proposed the creation of a “Patient Preference Predictor” (PPP), a computer-based algorithm designed to enhance—or, in some iterations of their framework, potentially replace—key aspects of the traditional surrogate decision-making process. The concept of the PPP relies on analyzing correlations between sociodemographic characteristics—such as age, gender, cultural background, religious beliefs, socioeconomic status, and prior healthcare experiences—and established healthcare preferences gathered from population-based studies. By synthesizing these data points, the model is intended to predict a specific patient’s unknown preferences with a higher degree of accuracy than traditional methods.

This proposal has garnered significant attention following Lamanna’s [5] suggestion that artificial intelligence (AI) and machine learning (ML) tools could be employed to analyze data from electronic health records (EHRs) and social media. Evidence indicates that algorithms analyzing Facebook “likes” alone can outperform spouses in predicting an individual’s personality traits [6]. Building on this, such technologies could potentially be utilized to estimate the confidence level in predicting whether a patient would consent to a specific treatment.

Since that time, although no functional prototype of a system capable of predicting patient preferences in clinical practice has been developed, a lively discussion has unfolded regarding both the technical viability and the ethical acceptability of implementing such a tool.

Recently, Earp et al. [7] introduced the Personalized Patient Preference Predictor (P4), a theoretical, individualized adaptation of the PPP that leverages advancements in ML. This approach employs tools such as fine-tuned large language models to process personalized data with greater efficiency and cost-effectiveness. Unlike the PPP, the P4 draws conclusions directly from patient-specific inputs, such as prior healthcare decisions, making it potentially more accurate and better attuned to the unique values and reasoning of each individual.

To date, no comprehensive analysis has been conducted to examine the arguments both for and against the use of artificial intelligence (AI)-based PPPs for incapacitated patients. This scoping review represents, to the best of our knowledge, the first attempt to map the discussion surrounding the application of AI-driven PPPs in clinical decision-making. By synthesizing the rationale and arguments presented in the academic literature thus far, this article aims to serve as a foundational reference for future research and innovation in this emerging field. The guiding research question for this study was as follows: what are the reasons for and against the use of AI-based PPPs in clinical decision-making?

## 2. Materials and Methods

This review provides a comprehensive cross-sectional analysis of the current debate surrounding the use of AI-based PPPs in clinical decision-making. It consolidates all the arguments that have been given in scholarly publications on the subject. The study was carried out and documented in accordance with the PRISMA-Ethics Reporting Guidelines [8], ensuring both transparency and methodological rigor.

A protocol was developed by three members of the author team (PR, DS, and AGS) prior to the initiation of the study and was subsequently approved by all researchers involved during a dedicated meeting on 8 July 2024. Given this thorough internal approval process, formal registration of the protocol was deemed unnecessary, as the researchers collectively ensured its rigor and compliance with methodological standards.

The search was conducted across three leading databases—PubMed, Web of Science, and Scopus—which were selected for their extensive and multidisciplinary coverage to ensure a thorough overview of the ongoing discussion.

The search strategy was structured around two semantic clusters: the “technical cluster”, which includes terms related to the technological basis of the applications, and the “preference prediction cluster”, which focuses on the patient preference aspect of these applications. To enhance the breadth of the search, equivalent terms and alternative spellings for key concepts were integrated into each cluster. The PubMed search strategy is presented in Table 1 and was adapted for each subsequent database, namely Web of Science and Scopus.

The search was limited to documents published in English. All database queries were performed in September 2024. Additionally, we carried out a manual search and reviewed the reference lists of all included studies to identify any further relevant publications.

The inclusion and exclusion criteria were established in advance (see Table 2). Publications were considered eligible if they explicitly focused on the use of PPPs, included a clear reference to the application of AI or ML, and presented at least one well-defined argument either supporting or opposing the use of PPPs. Consequently, studies that discussed PPPs without referencing AI or ML were excluded, along with those in which it was not possible to explicitly identify arguments either in favor of or against the use of PPPs.

Two blinded investigators (PR and CR) screened the studies using Rayyan software 2024 (https://www.rayyan.ai/, accessed on 26 December 2024). The software was used to detect and exclude duplicate studies, with each duplicate being manually reviewed by the two investigators. The investigators independently examined the titles and abstracts of the documents retrieved from the search that met the inclusion criteria. Disagreements were resolved through discussion, with unresolved cases brought to a third reviewer (AGS) for a final decision.

The full-text screening and analysis were conducted by PR and CR. Each study was reviewed at least twice prior to data extraction to ensure a comprehensive understanding of the content. The articles were examined for arguments both supporting (pros) and opposing (cons) the use of AI-based PPPs in clinical decision-making. To ensure accuracy, a third author (SM) cross-verified the extracted data against the original publications. Any discrepancies identified during this process were resolved by consulting the original study documents and engaging in discussions among the review team.

Given the well-known challenges of quality appraisal in the field of ethics and the absence of a universally accepted standard for evaluating ethical reasoning, we opted not to conduct a formal quality assessment of the studies. Additionally, some of the included studies are commentaries, making this type of analysis unfeasible.

The data synthesis was conducted using a narrative approach that was implemented in two iterative phases. Initial synthesis development: PR and CR independently created a textual summary for each study. Interpretation and findings: In the final stage, the identified themes were integrated into a coherent narrative aligned with the research question. PR and CR independently reviewed the thematic analysis and collaboratively refined the narrative synthesis.

## 3. Results

**Figure 1:**
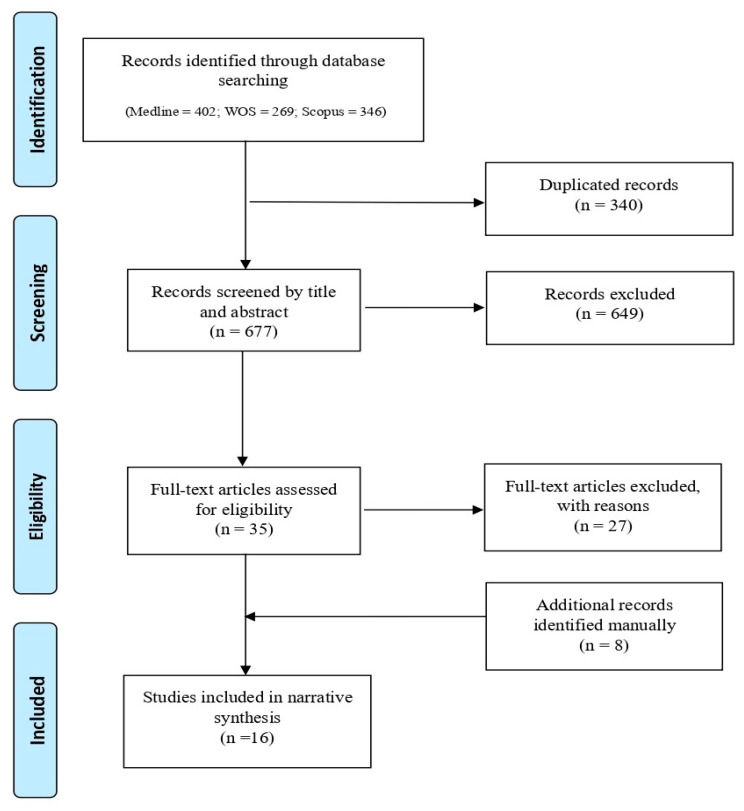
Prisma flow diagram—identification of relevant studies.

The search strategy yielded a total of 1017 records, from which 340 duplicates were removed. During the initial screening phase, 649 records were excluded because their titles and/or abstracts did not meet the eligibility criteria. A total of 35 full-text articles were then reviewed in the second-level screening, resulting in the selection of 8 articles for inclusion. An additional 8 articles were identified through reference screening, bringing the final count to 16 articles [5,7,9,10,11,12,13,14,15,16,17,18,19,20,21,22]. Details of the study selection process are illustrated in Figure 1. Prisma flow diagram—identification of relevant studies. The sample is evenly divided between journal articles (*n* = 8) and commentaries (*n* = 8), which were mostly published in the *Journal of Medical Ethics* (*n* = 10).

All arguments identified are detailed in the Supplementary Materials. They are presented objectively, irrespective of whether the authors of the respective publications support or oppose them. This approach ensures a balanced representation of the arguments, emphasizing their content as articulated in the original sources.

The arguments in favor are fewer in number compared to those against. The scientific community has identified multiple arguments potentially supporting the implementation of AI-PPPs in healthcare decision-making.

Similar tools hypothetically analyze vast datasets and provide highly accurate insights into patient preferences, potentially surpassing the predictive capabilities of human surrogates or family members [5,7,10,16]. By prioritizing patient-centered autonomy, AI-based PPPs may ensure that decisions align with individual values and claim to offer a truly personalized approach [5,7]. They could also alleviate the emotional burden on surrogates and family members who often face significant psychological strain when making life-or-death decisions for incapacitated patients [5,10,16].

Moreover, these predictors are designed to streamline the decision-making process, optimizing resource utilization by reducing the time and effort required to deliberate patient preferences. AI-based PPPs may also offer economic advantages by avoiding unnecessary treatments and reducing healthcare costs [5,10]. Their probabilistic assessments, such as those concerning resuscitation or organ donation, are intended to support human moral judgment by serving as a complement rather than a substitute [9,21].

Conversely, the scientific community has raised several objections and counterarguments regarding the use of AI-based PPPs. A recurring theme is the risk of bias reinforcement and amplification, as learning tools may perpetuate existing biases in healthcare data, potentially entrenching flawed processes and leading to inequitable outcomes [5,7,9,10,12,16]. This may be further exacerbated by concerns over sociodemographic generalizations, where assumptions about preferences based on group membership fail to capture individual complexities [7,9]. Moreover, reliance on statistical evidence risks oversimplifying the deeply personal and nuanced nature of healthcare decisions, neglecting the unique values and circumstances that influence individual choices [7,17,21].

Another recurring theme, as expected, concerns the potential violation of patient autonomy. Treating preferences as determined by statistical data tends to overlook the uniquely human capacity to form, revise, and justify preferences through reason [11]. The use of demographic data to predict treatment preferences may restrict individual freedom of choice, reducing autonomy to generalized patterns rather than personalized values [16]. Machines, by their nature, lack the ability to understand or engage with the underlying reasons and values that shape patient preferences [7,11,19,21], which can themselves be unstable [19]. Many individuals also possess strong meta-preferences (second-order preferences) that must be taken into account [13]. Additionally, patients may sometimes make choices that do not align with their core values or long-term preferences due to a variety of factors, such as emotional distress, fear, lack of understanding of their medical options, or the influence of external pressures from family, friends, or healthcare providers [14]. Moreover, when evaluating substituted decision-making, it is crucial to consider not only the patient’s treatment preferences but also their preferred decision-making processes, a factor that AI-based PPPs may fail to address [7].

Therefore, respecting patient autonomy requires more than accurate predictions based on population data or social media; it demands a deeper understanding of the individual’s unique values, reasoning, and the moral significance underlying their choices [7].

Failing to respect patient autonomy can lead to two significant risks. On the one hand, it may result in inauthenticity [22]; algorithm-driven decisions may lack authenticity, as they do not stem from the individual’s personal commitments and ideals, particularly in circumstances in which such values are important. On the other hand, there is a risk of AI systems dominating decision-making processes, leading to paternalism [18]. Furthermore, the use of AI may shift the balance of power, strengthening clinician authority at the expense of surrogates and family members, thereby diminishing their role in shared decision-making [16].

Further concerns pertain to privacy, data protection, and explainability. The use of sensitive patient information for algorithm training poses significant risks to confidentiality and raises questions about the security of these systems [9,20]. The lack of explainability and insufficient transparency in AI recommendations could undermine trust and lower the perceived value of the clinician’s (human) contribution [16].

A final concern is the potential dehumanization of decision-making, as excessive reliance on algorithmic outcomes risks diminishing the human element in patient care [5,10]. This reliance can erode the interpersonal and empathetic aspects that are essential in healthcare, reducing patient care to a purely mechanical process. By undermining the family’s role in shared decision-making, algorithms may marginalize the personal and relational dynamics that are vital for compassionate and context-sensitive choices [10]. Moreover, allowing AI systems to “extract” and “exploit” deeply personal experiences, such as suffering, introduces ethical risks that go beyond decision-making. This practice risks turning pain into a commodity as a resource and treating it as something to be monetized or operationalized rather than respecting it as a deeply personal and profound human experience. Such an approach not only diminishes the value of individual lived experiences but also prompts broader moral questions about the integration of AI into deeply human dimensions of healthcare [19].

## 4. Discussion

Summarizing the arguments for and against the use of AI-PPPs in healthcare decision-making, as identified in the literature, it can be observed that, on the one hand, these tools are supported for their potential to enhance the accuracy of predictions regarding patients’ preferences compared to human surrogates, alleviate the emotional burden on surrogates and family members, and optimize resource utilization. On the other hand, they are criticized for the risk of perpetuating existing biases in healthcare data, potentially violating patient autonomy, and raising concerns about privacy, data protection, and explainability, as well as posing a risk of dehumanizing the decision-making process.

By synthesizing the rationale and arguments presented in the academic literature thus far, this article could provide a foundational reference for future research and innovation in this emerging field. It lays the groundwork for a subsequent study we plan to undertake, which will examine the medico-legal challenges and implications of AI-PPP implementation in clinical practice.

Our paper could also provide critical insights for researchers and developers aiming to design and implement such tools, which, at this stage, remain largely conceptual. Furthermore, it could serve as a foundation for broader discussions on the multifaceted challenges associated with these technologies [23,24]. These include ensuring compliance with legal frameworks, safeguarding patient autonomy, addressing data protection and privacy concerns, and managing the risks of bias and opacity in AI systems. Engaging with these issues is essential not only to refine the technology itself but also to anticipate and address the regulatory and medico-legal complexities that would inevitably arise if such tools were implemented.

The identified topics appear to be closely aligned with many of the issues currently emerging in the broader debate surrounding the use of AI tools in healthcare [25,26]. These topics echo ongoing discussions about the ethical, legal, and social implications of integrating AI into medical practices, particularly in areas such as decision-making, patient autonomy, and data governance.

Finally, the identified arguments could serve as a valuable foundation for conducting qualitative research on the acceptability of AI-PPPs among the stakeholders involved in decision-making processes [27]. By exploring these perspectives, researchers can gain deeper insights into the perceptions, concerns, and expectations of patients, family members, clinicians, and other relevant stakeholders.

At present, to the best of our knowledge, only three qualitative studies have explored this subject, all of which include findings that align with our research. The first, conducted by Wendler et al. [4], examined whether patients support the use of PPTs in a sample of 1169 respondents. When asked if they would want a PPT integrated into the decision-making process in the event of incapacitation due to a car accident, 78.9% of respondents supported its use, while 15.2% opposed it. Those in favor highlighted the potential for PPTs to enhance the likelihood of receiving treatments consistent with their preferences and/or to lessen the burden on surrogate decision-makers.

The second study, led by Howard et al. [28], explored the perspectives of 26 experienced surrogates on the potential use of PPTs to support treatment decisions for incapacitated patients. Out of these participants, 21 were in favor of the use of PPTs, whereas 5 opposed them, arguing that decisions should prioritize the patient’s best interests rather than rely on substituted judgment. Key arguments included the belief that surrogates, rather than patients’ preferences, should guide decisions, alongside concerns that PPTs could be used to limit expensive treatments or introduce/perpetuate biases against minority populations.

Lastly, in the recent work conducted by Berzinger et al. [29], in which questionnaires were distributed to 200 German anesthesiologists and 200 German internists, physicians expressed significant concerns about AI-driven preference prediction, citing fears of losing individuality and humanity in decision-making, a lack of transparency in AI-generated outcomes, and doubts about AI’s capacity/ability to fully engage with ethical deliberations. However, they showed a more favorable attitude toward clinical ethics support services.

While these studies offer valuable initial insights, they also highlight the need for further empirical research to better understand how these tools are perceived and could be effectively implemented across diverse clinical and cultural contexts.

Overall, the discourse would greatly benefit from further in-depth research on the precision of surrogate decision-making in clinical contexts. Whether surrogates make choices that truly align with patients’ stated or inferred preferences remains an open question [3,30]. Surrogates’ predictive concordance fluctuates between randomness (50%) and near-perfect agreement (approaching 100%), yet the underlying determinants of this substantial variation remain unidentified and should be a central focus of forthcoming investigations [30].

Moreover, the disproportionate predominance of white participants from Western cultural backgrounds fails to capture the evolving demographic diversity of healthcare populations in industrialized nations. Future research should emphasize the inclusion of more heterogeneous samples, particularly by expanding racial and ethnic representation, encompassing a broader age spectrum of patients, and incorporating individuals with varying health conditions, including those in critical care [30].

Additionally, advancing this field requires the development of standardized, empirically validated tools for evaluating hypothetical treatment decision-making, as current instruments in this area remain scarce and inconsistently applied. Enhancing methodological rigor by integrating multimodal assessment frameworks—such as combining qualitative and quantitative approaches—would facilitate a more refined and holistic understanding of surrogate decision-making accuracy [30].

We acknowledge several limitations in the current review.

First, the framing of our research question may have unintentionally excluded some relevant studies that could have offered additional insights into the topic. The scope and boundaries defined for this review might have influenced the selection of included publications, potentially narrowing the breadth of perspectives analyzed.

Second, we relied on only three databases for our literature search, increasing the risk of overlooking pertinent studies. This limitation may have excluded publications that could have contributed valuable data or alternative viewpoints, thereby affecting the comprehensiveness and robustness of our findings.

Third, the publications included in this review predominantly reflect American and European perspectives. This lack of diversity limits the generalizability of our conclusions.

Future research should aim to address these gaps by broadening the search strategy, including a wider range of databases, and ensuring the inclusion of studies from underrepresented regions to capture a more global perspective.

## 5. Conclusions

Based on the findings of our review, the use of AI-PPPs in healthcare decision-making, as with most emerging technologies, has elicited both support and criticism. Proponents emphasize their potential to enhance the accuracy of predictions regarding patients’ preferences, mitigate the emotional strain on surrogates and family members, and optimize healthcare resource allocation. Critics, however, highlight risks such as reinforcing existing biases in medical data, undermining patient autonomy, raising critical concerns about privacy, data security, and explainability, and contributing to the depersonalization of decision-making processes.

Further empirical studies are necessary to evaluate the acceptability and feasibility of these tools among key stakeholders, including patients, surrogates, and clinicians. Moreover, robust interdisciplinary research is needed to explore the legal and medico-legal implications associated with their implementation, ensuring that these technologies align with ethical principles and support patient-centered and equitable healthcare practices.

## Figures and Tables

**Table 1:** Search strings.

Database	Search String
PubMed	(((predictor* OR prediction*) AND (preference*)) AND (patient*)) AND (((Artificial Intelligence) OR (Machine Learning) OR (clinical decision support) OR (ai advisory*)* OR (AI-driven decision support system) OR (Machine Learning-based clinical decision support)) OR ((algorithms[MeSH Terms]) OR (Artificial Intelligence[MeSH Terms]))
Web of science	((predictor* OR prediction *) AND preference* AND patient*) AND (“Artificial Intelligence” OR “Machine Learning” OR “clinical decision support” OR “ai advisory” OR “AI-driven decision support system” OR “Machine Learning-based clinical decision support” OR algorithms OR “Artificial Intelligence”)
Scopus	(( predictor* OR prediction*) AND preference* AND patient*) AND (“Artificial Intelligence” OR “Machine Learning” OR “clinical decision support” OR “ai advisory” OR “AI-driven decision support system” OR “Machine Learning-based clinical decision support” OR algorithms)

* indicates truncation to find all variants of a word.

**Table 2:** Inclusion and exclusion criteria.

Inclusion Criteria	Exclusion Criteria
▪ Focus on PPPs ▪ Explicit reference to AI or ML ▪ At least one well-defined argument either supporting or opposing the use of PPPs	▪ If the study focused on PPPs but did not include any reference to AI or ML ▪ If the argument in favor of or against PPPs was unclear

## Data Availability

Data supporting the reported results can be found in the Supplementary Materials.

## Supplementary Materials

The following supporting information can be downloaded at https://www.mdpi.com/article/10.3390/healthcare13060590/s1: File S1: Table of data extracted.
