# Supplementary material for: Should Artificial Intelligence-Based Patient Preference Predictors Be Used for Incapacitated Patients? A Scoping Review of Reasons to Facilitate Medico-Legal Considerations
Source: Healthcare (Basel). 2025 Mar 8;13(6):590. doi: 10.3390/healthcare13060590 (PMC11942106; doi:10.3390/healthcare13060590)
Supplement: Supplementary file 1 [file healthcare-13-00590-s001.zip › healthcare-3423549-supplementary.pdf]

File S1

Table of data extracted

Reference	Type of study	Aim of study	PROS	CONS
[5] Lamanna, C.; Byrne, L. Should Artificial Intelligence Augment Medical Decision Making? The Case for an Autonomy Algorithm. AMA J. Ethics **2018**, 20, E902–9105	Commentary	To explore how AI can leverage data from electronic health records and social media to predict the healthcare preferences of incapacitated individuals	- Enhanced accuracy: AI-driven predictors can analyze vast datasets to provide more precise insights into patient preferences.<br>- Reduced emotional burden: A computerized approach can alleviate the psychological strain on family members by supporting life-or-death decisions with data-driven recommendations.<br>- Patient-centered autonomy: The algorithm prioritizes and respects individual patient preferences, ensuring a truly patient-centric approach.<br>- Optimized resource utilization: Helps healthcare providers allocate resources more efficiently by reducing time and effort spent on preference-related deliberations.	- Risk of bias reinforcement: Learning tools may perpetuate existing biases and, if misapplied, could entrench an undesirable status quo.<br>- Dehumanization of decision-making: Over-reliance on algorithmic outcomes risks diminishing the human element in patient care and ethical deliberation.
[7] Earp, B.D.; Porsdam Mann, S.; Allen, J.; Salloch, S.; Suren, V.; Jongsma, K.; Braun, M.; Wilkinson, D.; Sinnott-Armstrong, W.; Rid, A.; Wendler, D.; Savulescu, J. A Personalized Patient Preference Predictor for Substituted Judgments in Healthcare: Technically	Article	To review recent advancements in AI research indicating that a P4 is technically feasible	- Enhanced performance: The (Personalized Patient Preference Predictor) P4 outperforms the PPP in accuracy, predictive capability, and upholding patient autonomy.	- Autonomy beyond prediction: Respecting patient autonomy involves more than accurately predicting treatment preferences based on population data; it requires understanding the individual's unique values and reasoning.<br>- Consideration of process preferences: Substituted decision-making should account not only for patients' treatment preferences but also for their preferred decision-making processes.<br>- Lack of value appreciation: The PPP (and presumably the P4) fails to respect an incapacitated individual's autonomy, as machines cannot comprehend the reasons and values underlying patient preferences.<br>- Evaluation challenges: Assessing the accuracy of a P4, surrogates, or the PPP poses significant difficulties due to the subjective and personal nature of preferences.<br>- Identity-based prediction: Predicting preferences requires a deep understanding of the patient's identity, which algorithms may lack.

Feasible and Ethically Desirable. Am. J. Bioeth. **2024**, 24, 13–26				- Implementation hurdles: Practical challenges arise in integrating a P4 into clinical practice, including workflow disruption and acceptance by clinicians and families.<br>- Inappropriate reliance: There is a risk of over-reliance on P4 outputs due to uncertainty about how much these predictions are grounded in plausible inferences from training data.
[9] Biller-Andorno, N.; Biller, A. Algorithm-Aided Prediction of Patient Preferences - An Ethics Sneak Peek. N. Engl. J. Med. **2019**, 381, 1480–14859	Article	To explore the ethical implications of training algorithms designed to predict individuals' advance health care decisions	- Probabilistic insights: Algorithms provide probabilities for preferences (e.g., resuscitation, organ donation), but must complement, not replace, human moral judgment.	- Individual complexity: Decisions in healthcare are deeply personal and may not be fully captured by generalized algorithms.<br>- Reinforcement of flawed processes: Algorithms risk perpetuating the consequences of prior poor decision-making.<br>- Bias amplification: AI systems may inadvertently reinforce existing biases in healthcare data.<br>- Data protection concerns: Use of sensitive patient information raises significant privacy and security challenges.
[10] Hubbard, R.; Greenblum, J. Surrogates and Artificial Intelligence: Why AI Trumps Family. Sci. Eng. Ethics **2020**, 26, 3217–322710	Article	To discuss the ethical issues surrounding whether an artificial agent should serve as a surrogate decision-maker	- Greater predictive accuracy: The algorithm is likely more precise in identifying the patient's preferred therapeutic options.<br>- Reduced proxy burden: It alleviates stress for family members and physicians by guiding treatment decisions.<br>- Economic efficiency: Avoids unwanted treatments, reducing unnecessary healthcare costs.<br>- Epistemic advantage: Algorithms often outperform proxies in accurately predicting patient preferences.<br>- Freedom from psychological biases: Unlike surrogates, algorithms are unaffected by stress, depression, or personal biases, leading to more reliable decisions aligned with the patient's values.	- Deeper familial insight: Families often have a more profound understanding of the patient's values and a stronger commitment to their well-being.<br>- Erosion of family autonomy: Relying on algorithms could significantly undermine the family's role in decision-making.<br>- Dehumanization risk: Dependence on algorithms may diminish the human element in patient care.<br>- Algorithmic bias: AI systems are vulnerable to biases that could lead to inequitable or inaccurate outcomes.
[11] Jardas, E.J.; Wasserman, D.; Wendler, D. Autonomy-Based Criticisms of the Patient Preference Predictor. J. Med.	Article	To review and evaluate autonomy-based criticisms of the PPP	- Comprehensive input: The Patient Preference Predictor (PPP) integrates all available factors to determine the patient's treatment preferences.	- Lack of public support: Many people oppose the use of a PPP in making treatment decisions during decisional incapacity.<br>- Preference for family autonomy: There is a belief that decision-making should rest with the family, not the PPP.<br>- Perceived loss of freedom: The PPP assumes limited individual freedom to make and change preferences.<br>- Questionable accuracy: The PPP may not significantly improve predictive accuracy in treatment decisions.

Ethics **2022**, 48, 304–31011				- Potential violation of autonomy: Treating preferences as determined solely by statistical data undermines the human ability to form, revise, and justify preferences based on reason.<br>- Use of non-endorsed reasons: The PPP may rely on factors that are not explicitly aligned with the patient's values or preferences.
[12] O’Neil, C. Commentary on ‘Autonomy-Based Criticisms of the Patient Preference Predictor’.12	Commentary	To analyze and respond to autonomy-based criticisms of the PPP		- Appropriate information basis: Predicting an individual’s preferences should rely on information they would consider relevant to shaping their personal choices or at least not reject as inappropriate.
[13] Mainz, J.T. The Patient Preference Predictor and the Objection from Higher-Order Preferences. J. Med. Ethics **2023**, 49, 221–22213	Commentary	To propose a new autonomy-based objection to the PPP		- Respect for second-order preferences: Many individuals have strong meta-preferences about how their preferences are predicted, which must be taken into account.
[14] Schwan, B. Sovereignty, Authenticity and the Patient Preference Predictor. J. Med. Ethics **2022**, 48, 311–31214	Commentary	To analyze and respond to autonomy-based criticisms of the PPP		- Inconsistent decision-making: Patients may make choices that do not align with their core values or long-term preferences.
[15] Earp, B.D. Meta-Surrogate Decision Making and Artificial Intelligence. J. Med. Ethics **2022**, 48, 287–28915	Commentary	To discuss the ethical issues surrounding whether an artificial agent should serve as a surrogate decision-maker		- Demographic generalizations: Belonging to a specific demographic group does not guarantee that an individual shares the same preferences as others within that group.

[16] Ferrario, A.; Gloeckler, S.; Biller-Andorno, N. Ethics of the Algorithmic Prediction of Goal of Care Preferences: From Theory to Practice. J. Med. Ethics **2023**, 49, 165–17416	Article	To introduce a novel way of analyzing the ethical concerns surrounding the PPP	- Personalized accuracy: AI methods like machine learning, proven effective in clinical settings, can generate personalized care predictions that are often more accurate than those of surrogates or family members.<br>- Emotional relief: High-performance AI algorithms can ease the emotional burden on surrogates tasked with making treatment decisions for incapacitated loved ones.	- Addressing biases and enhancing explainability: It is essential to mitigate biases in data collection, develop methods to improve algorithmic transparency, and ensure secure, reliable infrastructures for their use.<br>- Risk of limiting autonomy: Using demographic data as predictors for treatment preferences could restrict individual freedom of choice.<br>- Equity concerns: Predicting patient preferences with AI may raise equity issues, as care preferences often reflect diverse cultural norms or belief systems.<br>- Reinforcement of clinical authority: AI use might shift the balance of power, strengthening clinician authority over surrogates and family members.<br>- Trust and transparency challenges: The lack of explainability and insufficient transparency in AI recommendations could erode trust and diminish the perceived value of the clinician's human contribution.<br>- Overreliance on AI: The perceived credibility of AI recommendations may discourage clinicians from exercising independent judgment, reducing family involvement in shared decision-making and overly steering decisions toward AI-driven outcomes.
[17] Tretter, M.; Samhammer, D. For the Sake of Multifacetedness: Why Artificial Intelligence Patient Preference Prediction Systems Shouldn't Be for Next of Kin. J. Med. Ethics **2023**, 49, 175–17617	Commentary	To critically assess the ethical implications of AI-based PPP systems		- Recognition of multifaceted individuality: Shared decision-making must account for individuals as complex personalities with diverse desires and goals. However, since AI systems often reflect only one dimension and may incorporate biased data, and clinicians typically lack deep personal knowledge of the patient, only family members can be considered truly suited for this task.
[18] Diaz Milian, R.; Bhattacharyya,	Commentary	To underscore the importance of the		- Risk of AI paternalism: AI systems could dominate decision-making processes, potentially overriding individual preferences and limiting autonomy.

A. Artificial Intelligence Paternalism. J. Med. Ethics **2023**, 49, 183–18418		implementation of safeguards for AI algorithms before they are deployed in clinical practice		
[19] Jesudason, E. Fracking Our Humanity. J. Med. Ethics **2023**, 49, 181–18219	Commentary	To discuss the ethical issues surrounding whether an artificial agent should serve as a surrogate decision-maker		- Instability of human preferences: Human preferences are dynamic and context-dependent, making them challenging to capture accurately.<br>Ethical concerns over exploitation: Allowing AI to "extract" and "exploit" deeply personal experiences, such as suffering, risks commodifying pain as a resource, akin to "the new oil," treating it as something to be monetized rather than respected.
[20] Sharadin, N. Predicting and Preferring. Inquiry **2023**, 1-1220	Article	To examine the moral implications of using ML models to predict incapacitated patients' treatment preferences, highlighting the need for ethical assurances before proceeding with experimental research		- Logistical challenges: Legally acquiring the necessary data poses significant obstacles, including compliance with privacy regulations and ethical considerations.<br>Risk of performative predictions: AI may generate predictions that appear accurate or insightful but lack meaningful alignment with the patient's true preferences or values.
[21] Makins, N. Algorithms Advise, Humans Decide: The	Article	To introduce a distinction that has been largely	- The PPP should be considered an evidential tool that supports human decision-making by predicting patient preferences, rather than determining which options to pursue.	- Bare statistical evidence: Reliance on statistical data alone may oversimplify the complexity of individual patient preferences.

Evidential Role of the Patient Preference Predictor. J. Med. Ethics **2024,** 21		overlooked in debates about the PPP – the differentiation between algorithmic prediction and decision-making – clarifying their ethical and practical implications in surrogate decision-making		- Value of surrogate decision-making: There are compelling reasons to keep decision-making with surrogates, beyond their ability to accurately reflect the patient's preferences, such as emotional and relational factors.<br>- Objection of endorsed reasons: Decisions based on AI outputs may not align with the endorsed reasons or values that truly matter to the patient.
[22] Karlan, B. Authenticity in Algorithm-Aided Decision-Making. Synthese **2024**, 204, 9322	Article	To present a framework for algorithm-aided decision-making that can lead to inauthenticity		- Inauthenticity of algorithm-aided decisions: Decisions guided by algorithms may lack authenticity, as they do not arise from the individual's own commitments and ideals, particularly in domains where such values are explicitly held.
